# Quinazolin-derived myeloperoxidase inhibitor suppresses influenza A virus-induced reactive oxygen species, pro-inflammatory mediators and improves cell survival

**DOI:** 10.1371/journal.pone.0254632

**Published:** 2021-07-19

**Authors:** Juan A. De La Cruz, Thota Ganesh, Becky A. Diebold, Weiping Cao, Amelia Hofstetter, Neetu Singh, Amrita Kumar, James McCoy, Priya Ranjan, Susan M. E. Smith, Suryaprakash Sambhara, J. David Lambeth, Shivaprakash Gangappa

**Affiliations:** 1 Influenza Division, Centers for Disease Control and Prevention, Atlanta, Georgia, United States of America; 2 Department of Pharmacology, Emory University, Atlanta, Georgia, United States of America; 3 Department of Pathology, Emory University, Atlanta, Georgia, United States of America; University of Messina, ITALY

## Abstract

Superoxide radicals and other reactive oxygen species (ROS) are implicated in influenza A virus-induced inflammation. In this *in vitro* study, we evaluated the effects of TG6-44, a novel quinazolin-derived myeloperoxidase-specific ROS inhibitor, on influenza A virus (A/X31) infection using THP-1 lung monocytic cells and freshly isolated peripheral blood mononuclear cells (PBMC). TG6-44 significantly decreased A/X31-induced ROS and virus-induced inflammatory mediators in THP-1 cells (IL-6, IFN-γ, MCP-1, TNF-α, MIP-1β) and in human PBMC (IL-6, IL-8, TNF-α, MCP-1). Interestingly, TG6-44-treated THP-1 cells showed a decrease in percent cells expressing viral nucleoprotein, as well as a delay in translocation of viral nucleoprotein into the nucleus. Furthermore, in influenza A virus-infected cells, TG6-44 treatment led to suppression of virus-induced cell death as evidenced by decreased caspase-3 activation, decreased proportion of Annexin V^+^PI^+^ cells, and increased Bcl-2 phosphorylation. Taken together, our results demonstrate the anti-inflammatory and anti-infective effects of TG6-44.

## Introduction

Several cellular enzymes including NADPH oxidase (Nox), nitric oxide synthase (NOS), cytochrome P450 reductase, myeloperoxidase (MPO), and xanthine oxidase are involved in generation of superoxide radicals in different cell types [1, 2]. Earlier studies identified reactive oxygen species (ROS)-generating enzymes in several cell types of the human airway as a critical component of local host defense [1, 3]. ROS produced by Nox enzymes can lead to increased phosphorylation of NF-κB [4] and can induce expression of pro-inflammatory mediators such as TNF-α, MCP-1 [5, 6], and IL-6 [7]. Moreover, ROS, when produced in excess by leukocytes, can exert cytotoxicity and lead to increased tissue damage, especially in the lung tissue [8–10].

Several studies have examined the role of ROS and ROS-generating enzymes in influenza A virus infection. Oda et al. showed increased superoxide anion (O_2_^-^) production by alveolar phagocytic cells obtained from influenza A virus (H2N2)-infected mice [11]. Imai et al. established an increase in ROS levels in human peripheral blood mononuclear cells (PBMC) treated with inactivated H1N1 and H5N1 influenza A virus subtypes [9]. In addition, other studies addressing the role of ROS generating enzymes on disease severity caused by influenza A virus found increased ROS levels in response to infection [8, 12, 13]. Furthermore, in mouse model of influenza infection, studies focused on suppression of ROS using ROS scavenger compounds showed improvement in influenza A virus-induced disease severity [8, 11, 12, 14–16]. In these studies, different types of ROS-inhibitors such as pyran-polymer conjugated super oxide dismutase (SOD), NG-monomethyl-l-arginine, ebselen, apocyanin, Glycyrrhizin, and recombinant human catalase were evaluated.

Quinazolin derivatives, a class of N-containing heterocyclic compounds, have shown an array of biopharmaceutical activities including anti-oxidant [17], anti-inflammatory [18], anti-tumor [19, 20], and anti-bacterial [21, 22] properties, in a variety of experimental models. In the present study, we anticipated that these anti-oxidant and anti-inflammatory characteristics would be demonstrated by TG6-44, a thioxo-dihydroquinazolin-one derivative [23], in a model of influenza infection. Notably, in our earlier studies, TG6-44 (Compound 1c) was shown to inhibit MPO-specific ROS generation (85% inhibition with an IC_50_ of 0.8 ± 0.1 μM) in a neutrophil membrane cell-free system in a NADPH-dependent L-012 chemiluminescence assay [23].

In the current study, we evaluated TG6-44 for ROS inhibition in an *in vitro* model of influenza A virus infection. We observed increased ROS levels in lung monocytic cells (THP-1) infected with influenza A (A/X31) virus accompanied by an increase in inflammatory mediators and virus-induced cell death, all of which were suppressed by treatment with TG6-44. Many of these effects were also observed in freshly isolated PBMC. These results demonstrate that TG6-44 has anti-inflammatory activity in influenza-virus infected cells *in vitro*, suggesting that this compound, or similar quinazoline derivatives, may prove beneficial as adjunct therapeutics during influenza A virus infection.

## Materials and methods

### Cell culture

THP-1 (human monocytic leukemia cell line; ATCC-TIB202) cells were maintained in RPMI 1640 (Invitrogen). Madin-Darby canine kidney (MDCK, ATCC-CRL2936) cell line was maintained in Dulbecco’s modified Eagle’s medium (DMEM, Invitrogen) supplemented with 10% fetal bovine serum (FBS), 100U/mL penicillin, 100μg/mL streptomycin, and 2mM L-glutamine. Whole blood was collected from healthy volunteers by obtaining written and informed consent under a protocol (CDC-IRB #1652) approved by the CDC’s Institutional Review Board, and PBMC were isolated by centrifugation through Ficoll-Hypaque solution.

### Virus infection and TG6-44 treatment

THP-1 cells (2x10^6^) were re-suspended in 200μL virus infection media (cell culture media supplemented with 0.3% BSA). Cells were either mock infected with PBS or infected with A/X31, a commonly used H3N2 subtype of influenza A virus [24], at multiplicity of infection (MOI) of 0.1 or 1.0, for 1 h at 37˚C. A/X31 stock was generated as described earlier [25] by harvesting allantoic fluid from 10-day-old, virus inoculated, embryonated eggs and determining virus titer (pfu; plaque forming units) by standard plaque assay [26]. After adding virus, some samples were treated with TG6-44 (10 or 50 μM in 10μl), a quinazolin derivative (6-chloro-3-(3-(dimethylamino)propyl)-2-thioxo-2,3-dihydroquinazolin-4(1H)-one hydrochloride) identified, synthesized, and characterized as a MPO-specific ROS inhibitor (Compound 1c) at the Department of Pathology, Emory University, through high-throughput screening (HTS) approach [23], dissolved in vehicle of 35% polyethyl-glycol (PEG, Sigma), 10% ethanol (Sigma) and 55% ultrapure water (Gibco). As discussed below, in some experiments, TG6-44 and A/X31 were either briefly incubated together (10 min, 37˚ C) prior to infection or TG6-44 treatment was delayed 2 h relative to A/X31. After 1 h incubation, cells were washed 3 times and plated in 2 mL of media in a 6-well plate. In the case of 24 h treatments, TG6-44 treatments were repeated every 8 hours.

### Virus titer

Virus titer was determined using standard plaque assay [26]. Briefly, confluent monolayers of MDCK cells in 6-well plates were washed with media (supplemented with 0.3% BSA) followed by addition of serial 10-fold dilutions of virus-containing cell culture supernatants and incubation for 1 h at 37˚C. Next, cells were washed with virus infection media and overlayed with 1.0% agarose (Lonza) mixed 1:1 with 2X L15 medium (Lonza) containing 4mM 4-(2-hydroyethyl)1-piperazineethanesulfonic acid (HEPES), 2mM L-glutamine, 5μg/mL gentamycin, and 1.5mg/mL sodium bicarbonate. To the mix, 1μg/mL of N-P-tosyl-1-phenylalanine chloromethyl ketone (TPCK)-treated trypsin was added (Sigma). Plaques were counted after fixing and staining the cell monolayer with 0.3% crystal violet solution (BD) after 72 h incubation.

### Flow cytometry

Apoptosis was detected using Annexin V apoptosis detection kit FITC (e-Bioscience; catalog number 88-8005-72) per manufacturer’s instructions. Briefly, after harvesting THP-1 and PBMC at the indicated times, cells were centrifuged and washed once with cold PBS followed by another wash with 1X cold Annexin V Binding Buffer. To the cells (10^6^ cells/100μl), 5μL of fluorochrome-conjugated Annexin V was added and incubated in the dark for 15 min. Cells were washed once with 1X Annexin V Binding Buffer and re-suspended in 300μL of 1X Annexin V Binding Buffer containing 5μL Propidium iodide (eBioscience). Percent apoptosis in PBMC subsets (T cells, B cells, dendritic cells, and monocytes) was determined by staining with antibodies (BD Bioscience) specific for immune cell surface markers: HLA-DR-allophycocyanin-Cy7 (Catalog number 335796), CDllc-APC (Catalog number 340544), CD19-PerCP (Catalog number 340421), CD14-Alexa Fluor 700 (Catalog number 557923), CD3-PE-Cy7 (Catalog number 563423). Samples were analyzed using an LSRII flow cytometer (BD Bioscience).

### PCR array

Total RNA was extracted from infected and control samples using TRIzol (Invitrogen) as described by the manufacturer. Total RNA (200ng) was reverse transcribed by the addition of 5X reverse transcriptase buffer (Roche), oligo (dT) primer (Invitrogen), dNTP (Invitrogen), random hexamers (Invitrogen), reverse transcriptase (Roche) and water to the RNA in a 20μL volume reaction. Thermocycler conditions for cDNA synthesis were 48˚C for one hour and 85˚C for 10 minutes. Real-time PCR was carried out using SYBR Green PCR Master Mix (Invitrogen) per manufacturer’s instructions. Twenty-five μL of the SYBR Green PCR Master Mix/cDNA was added to each well of a 96-well PCR array plate (SABioscience, PAHS-012-A-12, RT^2^ Profiler PCR Array Human Apoptosis). Thermocycler conditions were 1 cycle at 95˚C for 10 min, 40 cycles at 95˚C for 30 sec and 60˚C for 1 min and 1 cycle at 95˚C for 1 min, 55˚C for 30 sec and 95˚C for 30 sec using Stratagene MX3005P thermocycler (Agilent Technologies).

### Real-time PCR

The amount of cDNA synthesized from 200 ng total RNA, using the protocol described above, was used in the following reaction: 2X SYBR Green Reaction Mix (12.5 μL), SuperScript III RT/Platinum *Taq* mix (1 μL) (Invitrogen), forward primer [1 μL (10 μM)], and reverse primer [1 μL (10μM)] in a 25 μL reaction for IFN-β and β-actin. IFN-β forward- CAACTTGCTTGGATTCCTACAAAG; IFN-β reverse- TATTCAAGCCTCCCATTCAATTG; β-actin forward ACCAACTGGGACGACATG GAG AAA; β-actin reverse TAGCACAGCCTGGATAGCAACGTA. Cycle conditions were: 1 cycle at 48˚C for 15 min, 1 cycle at 95˚C for 10 min, 40 cycles at 95˚C for 30 sec, 60˚C for 1 min, and 95˚C for 1 min, 1 cycle at 95˚C for 1 min, 55˚ for 30 sec, and 95˚C for 30 sec.

### Confocal immunofluorescence

Following treatment, cells were pelleted, washed with cold PBS, fixed with 4% v/v paraformaldehyde in PBS, permeabilized with 0.1% Triton X-100 in PBS and blocked with 1% BSA and 5% normal goat serum in PBS. Cells were stained with mouse anti-Influenza A-virus-nucleoprotein (1:2500) overnight, followed by goat anti-mouse Alexa Fluor Flour 488 (1:5000, Molecular Probes) for 2h. Cells were mounted on slides with Prolong Antifade Mounting Media (Molecular Probes) containing DAPI to visualize nuclei. Images were captured using a LSM 710 inverted confocal microscope (Zeiss, Oberkochen, Germany) equipped with a x100 numerical aperture oil immersion objective. Images were processed using identical parameters and assembled into final figures using Abode Photoshop CS3 (Adobe Inc).

For calculating influenza nucleoprotein (NP)-positive cells, five independent 20x-image fields from each condition were evaluated. The total numbers of NP-positive cells was divided by the total numbers of DAPI-positive cells in each field to estimate the percent NP-positive cells (n = >500). For calculating cells expressing NP in the nucleus or cytoplasm, NP-positive cells (n = 50) were evaluated for nuclear or cytoplasmic expression of NP. Cells expressing NP in the cytoplasm or nucleus was divided by the total NP-positive cells counted in each field to estimate the percent expression in nucleus or in the cytoplasm.

### Western blot

Cells washed with cold 1X PBS were lysed using 1X RIPA lysis buffer (Cell Signaling) containing a cocktail of protease inhibitors (Roche Diagnostics). Equal amounts of protein were loaded on to a 4–12% Bis-Tris Criterion XT precast gel (Biorad). Primary antibodies for Bcl-2, phosphorylated Bcl-2 (ser70) (Cell Signaling, Catalog number 2871), and Caspase3 (Cell Signaling, Catalog number 9661S), were all used at a dilution of 1:1000. Anti-rabbit secondary antibody (Cell Signaling, Catalog number 7074S) was used at 1:5000. Influenza A-virus- NP antibody was used at a dilution of 1:5000 and β-actin (Sigma, Catalog number A2228) antibody was used at 1:10000. Antibody dilution buffer and blocking buffer was 5% BSA in 1X PBS-0.1% Tween. Images were acquired with Kodak Image Station 400MM Pro (Molecular Imaging Systems) using Pico luminescence HRP detection kit (Thermo Scientific).

### ROS assay

Cells washed in room temperature HBSS with calcium and magnesium (Gibco) were added to 96-well plates at 2x10^4^ cells/150μL and allowed to equilibrate for 20 min at 37˚C. After the incubation period, PMA (50μM), diphenyleneidonium chloride (DPI) (10μM) (Sigma), SOD (5 Units) (MP Biomedicals), and/or TG6-44 (50 μM) were added to infected and control cells. L-012 (Wako) was added at a concentration of 50μM followed by 0.3 units of horseradish peroxidase (Sigma). Plates were immediately read using a BioTek plate reader set for continuous reading every minute for 80 min at 37˚C.

### Bio-Plex

Following the manufacturer’s protocol, a customized panel of 8 inflammatory cytokines and chemokines (IL-6, IL-8, INF-γ, IP-10, MCP-1, MIP-1β, RANTES and TNF-α) was measured using a Bio-Plex suspension array system (Bio-Rad). Briefly, cell culture supernatants and assay standards were added to a 96-well filter plate, followed by anti-cytokine-coupled beads, biotinylated bead detection antibodies, and PE-conjugated streptavidin. The plate was read using a Bio-Plex suspension array system and data were analyzed using Bio-Plex Manager 4.0 software (Bio-Rad) to calculate protein concentrations (pg/mL) based on standard curves.

### Statistical analysis

Statistical analysis was performed using GraphPad Prism 5.0 software (GraphPad Software). A Student’s *t* test was used to analyze differences in measures of ROS, inflammatory mediators, virus titer, and cell death. The data are presented as mean ± SEM. All differences were considered statistically significant when the *p-*value was ≤0.05.

## Results

### TG6-44, a small molecule inhibitor of ROS, suppresses virus-induced ROS in A/X31-infected THP-1 cells and PBMC

**Fig 1:**
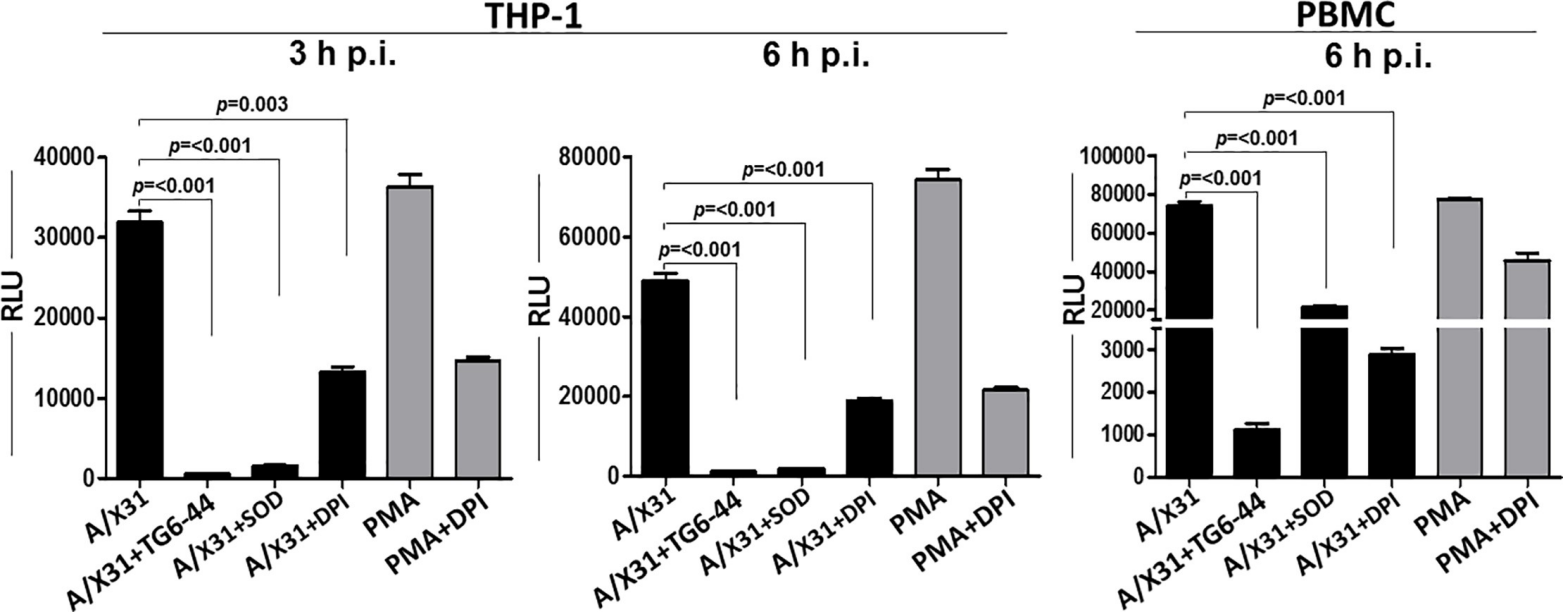
TG6-44 treatment decreases A/X31-induced ROS in THP-1 cells. A/X31-infected and PMA (50 μM)-treated THP-1 cells and PBMC incubated with TG6-44 (50μM), DPI (10 μM), or SOD (5 Units) were analyzed at 3 h and 6 h post-treatment for ROS levels, as described in Materials and Methods. Data represent results from one of three independent experiments. Values represent mean ± SEM.

Previous demonstration of the impact of ROS inhibition on influenza infection [8, 11, 12, 14, 15] prompted us to investigate the effects of TG6-44, a small molecule ROS inhibitor, on virus-induced ROS. TG6-44 is a quinazolin derivative developed in our laboratory by a HTS approach for small molecule inhibitors of ROS [23]. Previous studies demonstrated inhibition of MPO-mediated ROS generation by TG6-44 (85% inhibition with an IC_50_ of 0.8 ± 0.1 μM) in a neutrophil membrane cell-free system in a NADPH-dependent L-012 chemiluminescence assay [23]. In our current studies, in response to influenza A virus (A/X31) infection, THP-1 cells exhibited an increase in ROS production as early as 3 h p.i. (Fig 1, left panel). This increase in ROS production caused by influenza A virus infection has also been observed with a different subtype of influenza A virus and cell type [9]. As a positive control, ROS production by THP-1 cells was markedly stimulated by treatment with PMA, an activator of protein kinase C that phosphorylates and activates p47^*phox*^ of Nox2 in neutrophils and monocytes [27, 28]. ROS level decreased significantly in A/X31-infected cells treated with TG6-44 (50 μM). As expected, ROS levels decreased significantly in infected cells treated with DPI, an inhibitor of flavoenzymes, or by SOD, an enzyme that catalyzes O_2_^-^ into oxygen and hydrogen peroxide [29, 30]. Induction of ROS by virus infection, and its reduction by TG6-44, as well as DPI and SOD treatments, was also apparent at 6 h (Fig 1, middle panel). Similarly, TG6-44 mediated suppression of virus-induced ROS was detectable at 6 h p.i. in A/X31-infected human PBMC (Fig 1, right panel).

### TG6-44 treatment decreases virus-induced inflammatory mediators in influenza A virus-infected cells

**Fig 2:**
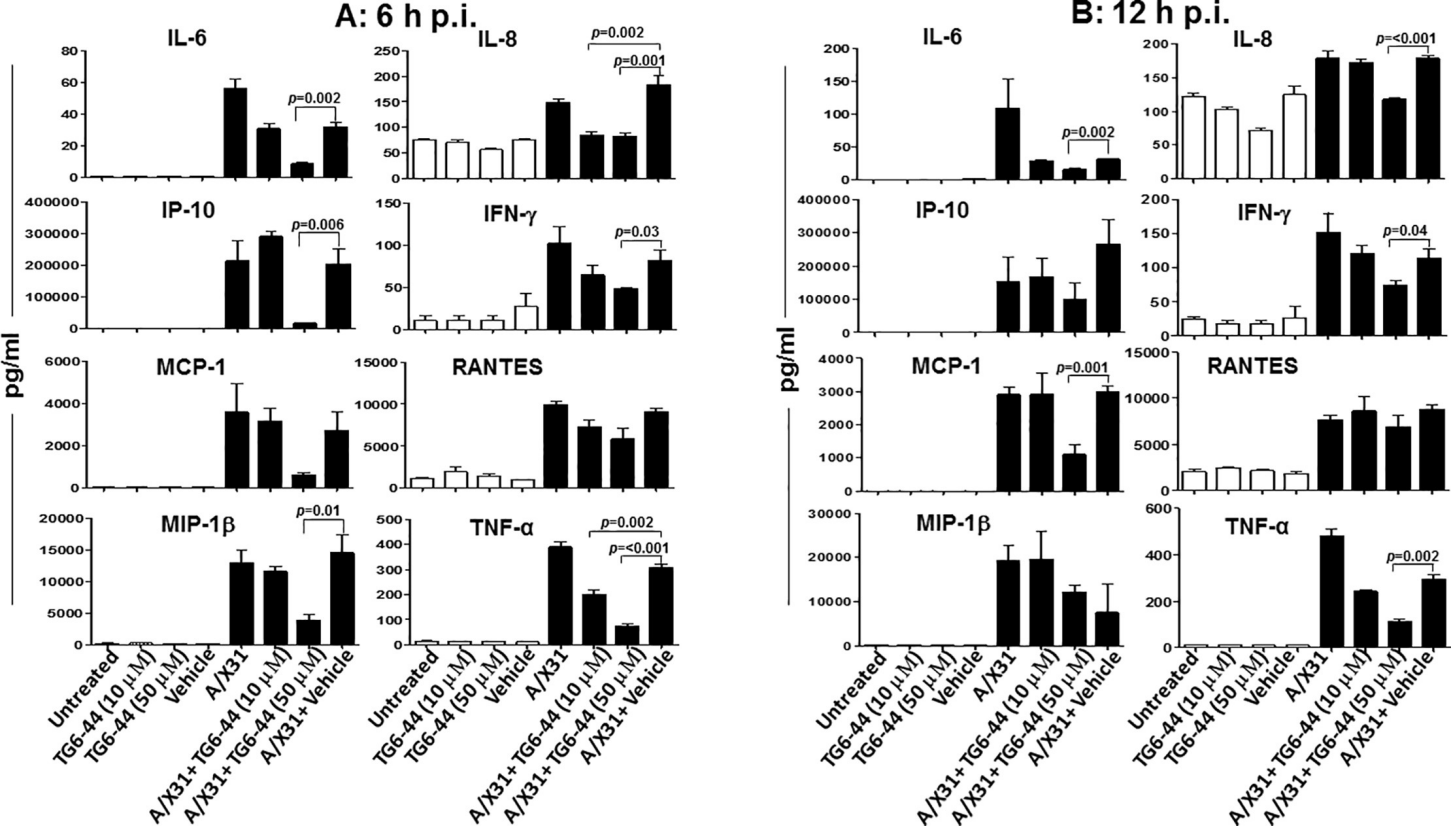
TG6-44 treatment leads to suppression of A/X31-induced inflammatory mediators in THP-1 cells. Cell culture supernatants from A/X31-infected THP-1 cells treated with either vehicle and/or TG6-44 were harvested at 6 h (A) and 12 h (B) p.i. and assayed for inflammatory mediators by Bio-Plex assay, as described in Materials and Methods. Data represent results from four independent experiments. Values represent mean ± SEM.

Since ROS is expressed in THP-1 cells in response to influenza virus infection (Fig 1) and, more importantly, is associated with enhanced inflammation and influenza A virus-induced disease severity [8, 11, 14], we examined the effects of TG6-44 on influenza A virus-induced inflammatory mediators. A/X31-infected THP-1 cells were treated with two different concentrations of TG6-44 (10 or 50μM). The two concentrations used were non-toxic to THP-1 cells as assessed by flow cytometry (S1 Fig). Untreated cells and cells treated with vehicle were used as controls. TG6-44 was added to the cell cultures and replenished every 8 hours until 24 hours p.i. Cell culture supernatants harvested at 6, 12, and 24 h p.i. were assayed for virus-induced inflammatory mediators. As shown in Fig 2, by 6 h p.i. (Fig 2A), A/X31-infected THP-1 cells treated with 10 μM TG6-44 showed a significant reduction in virus-induced IL-8 and TNF-α, when compared to non-treated A/X31 infected cells. Interestingly, THP-1 cells treated with a higher concentration of TG6-44 (50 μM) showed suppression of additional inflammatory mediators (Fig 2A; IL-6, IP-10, MIP1-β, IFN-γ). The suppressive effect of 50 μM TG6-44 treatment on virus-induced inflammatory mediators (IL-6, IL-8, IP-10, IFN-γ, MCP-1, and TNF-α) was also apparent in supernatants harvested at 12 h p.i. (Fig 2B). By 24 h p.i, even though suppressive effects of 50 μM TG6-44 were seen with some inflammatory mediators (IL-6, MIP1-β, TNF-α, and RANTES), the levels of 3 inflammatory mediators (IP-10, MCP-1, and IFNγ) were significantly increased (S2A Fig). Notably, neither of the 2 concentrations of TG6-44 treatment had any influence on the basal levels of inflammatory mediators of uninfected THP-1 cells (Figs 2A, 2B and S2A). Also, as shown in S2B Fig, when PBMC were used for this experiment, treatment with TG6-44 at 50 μM led to a significant reduction in the levels of inflammatory mediators IL-6, MCP-1, RANTES, and TNF-α by 12 h p.i.

### TG6-44 treatment decreases X31-infectivity in THP-1 cells

**Fig 3:**
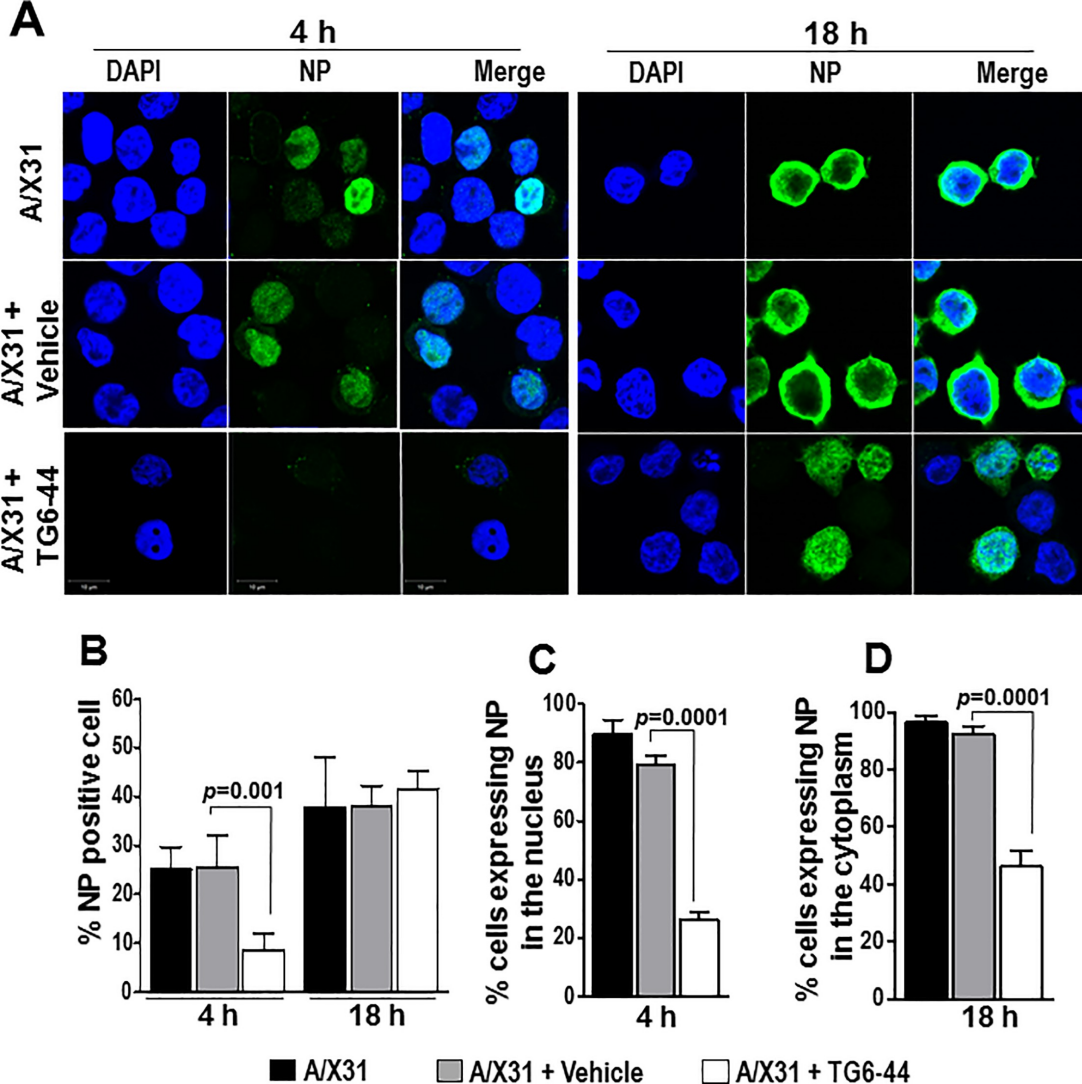
TG6-44 treatment leads to a decrease in NP-positive cells and altered expression patterns of NP in A/X31 infected THP-1 cells. (A) Confocal fluorescence microscopy images evaluating localization patterns of NP are shown. Images were acquired from A/X31 infected THP-1 cells treated with vehicle and/or TG6-44 (50 μM) and harvested at 4 and 18 h p.i. Cell nuclei are shown as blue and NP as green. Scale bar corresponds to 10 μm. (B) Quantitative morphometric analysis showing the proportions of NP-positive cells in A/X31-infected THP-1 cells in the presence of vehicle or TG6-44. Values are the means ± S.D. of five 20x-image fields from one of two independent experiments. (C) Quantitative morphometric analysis showing the proportions of NP-positive cells expressing NP in the nucleus at 4 h p.i. with A/X-31, in the presence of TG6-44 and/or vehicle. Results are expressed as means ± S.D. (D) Quantitative morphometric analysis showing the proportions of NP-positive cells expressing NP in the cytoplasm at 18 h p.i. with A/X31 in the presence of TG6-44 and/or vehicle. Results are expressed as means ± S.D.

**Fig 4:**
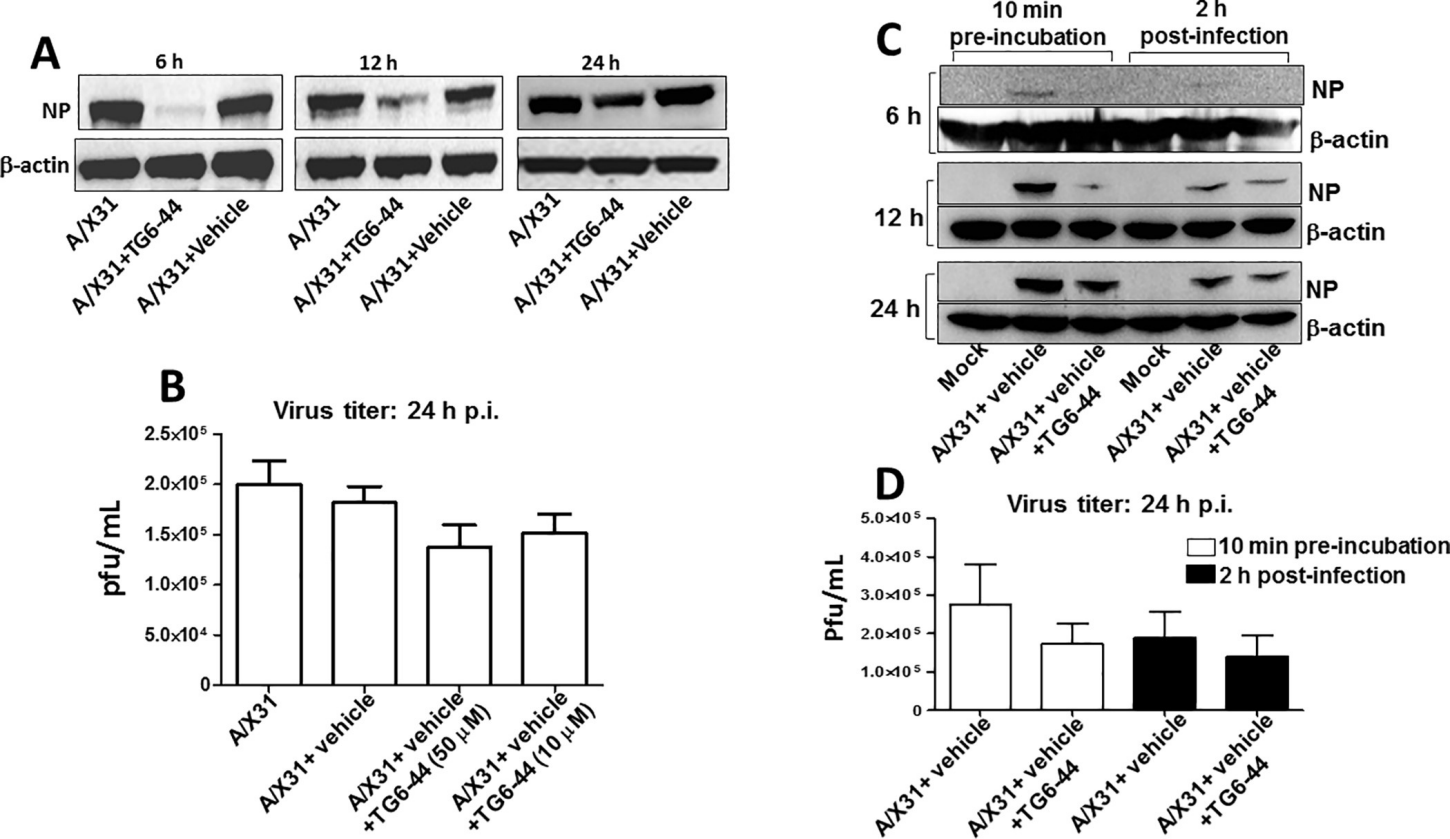
TG6-44 treatment changes kinetics of virus infectivity in THP-1 cells. (A) and (C); Cell lysates prepared from A/X31-infected cells treated with either vehicle or TG6-44 (50 μM) were analyzed for NP protein expression and β-actin, by western blot assay, at 6, 12, and 24 h p.i., as described in Materials and Methods. Data represent results from one of three independent experiments. (B) and (D); Cell culture supernatants from A/X31-infected cells treated with vehicle and/or TG6-44 (10 or 50 μm) were analyzed for virus titer at 24 h p.i by plaque assay, as described in Materials and Methods. Values in panel (B) and (D) represent mean ± SEM from three independent experiments.

**Fig 5:**
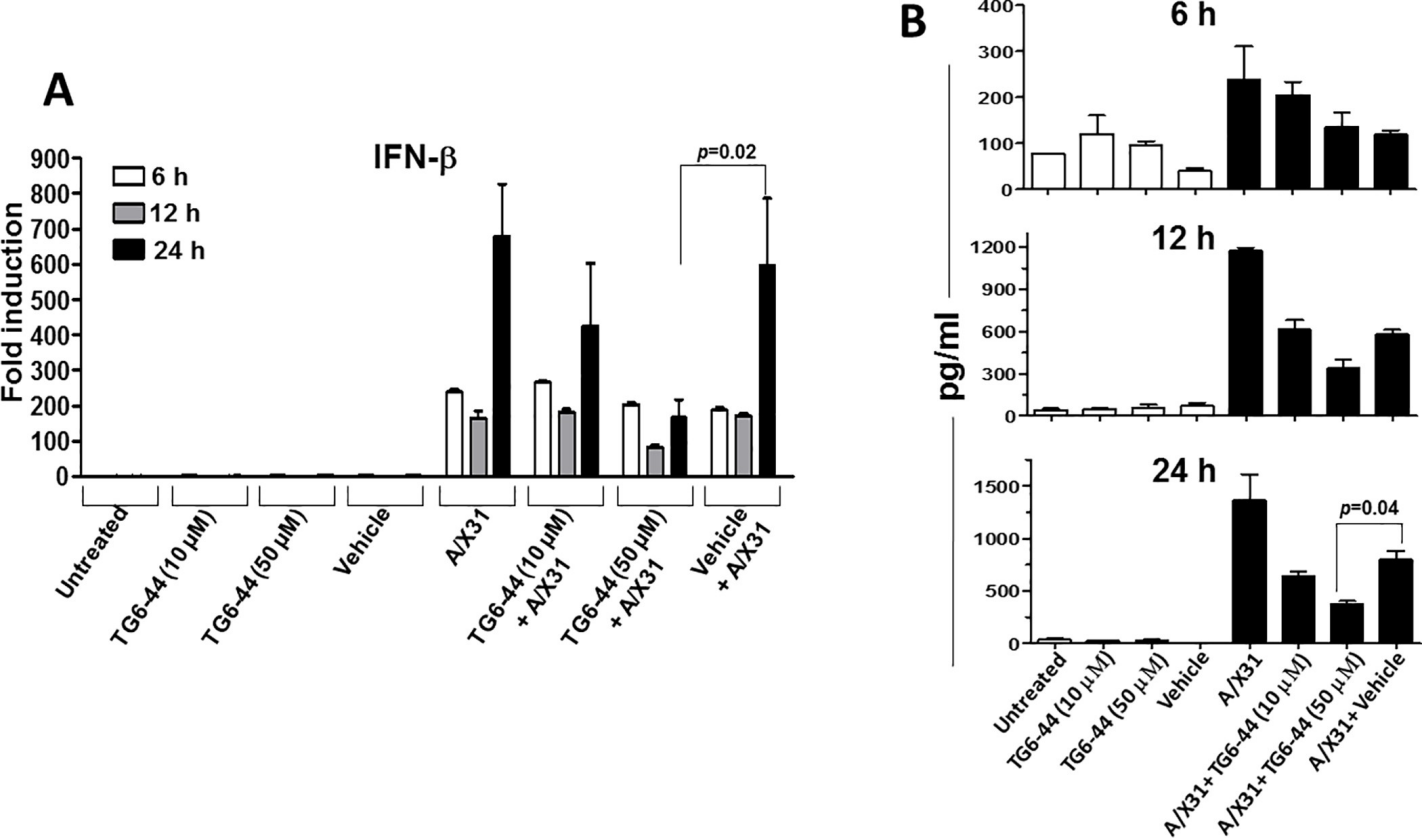
TG6-44 treatment decreases IFN-β levels in A/X31-infected THP-1 cells. Cell cultures from untreated, mock-treated, and/or TG6-44-treated A/X31-infected THP-1 cells were analyzed for IFN-β mRNA in cell lysates (A) and IFN-β protein in cell culture supernatants (B) at 6, 12, and 24 h p.i., as described in Materials and Methods. A represents results from three independent experiments. B represents results from one of three independent experiments. Values represent mean ± SEM.

Previous studies using ROS inhibitors to suppress influenza A virus-induced ROS in a mouse model showed suppression of lung inflammation as well as virus titer in the lung tissue [12, 31]. Having found in this study that TG6-44 treatment suppressed influenza A virus-induced ROS and inflammatory mediators in THP-1 cells and PBMC (Figs 1, 2 and S2), we next examined its effects on virus infectivity in THP-1 cells. As a measure of virus infectivity, we determined the viral nucleoprotein (NP) positive cells by confocal microscopy in A/X31 infected THP-1 cells treated either with vehicle or TG6-44. We found that percent NP expressing cells by 4 h p.i were significantly reduced in TG6-44-treated cells (Fig 3A, left panel and Fig 3B). However, by 18 h p.i, percent NP positive cells in the A/X31 infected cells were comparable between vehicle treated and TG6-44 treated cell cultures (Fig 3A, right panel and Fig 3B, left panel). Next, we determined the localization of NP at 4 h and 18 h p.i. At 4 h p.i, as shown previously by Neumann et al. [32], NP was seen predominantly in the nucleus of A/X31-infected cells (Fig 3A, left panel). Addition of vehicle to the cells did not alter localization of NP in A/X31-infected cells. However, NP expression was restricted primarily to cytoplasm of A/X31-infected cells in the TG6-44-treated THP-1 cells (Fig 3A, lower panel and Fig 3C). At 18 h p.i, while majority of NP expression in A/X31 and vehicle-treated cells was re-localized to cytoplasm as expected [32], TG6-44 treated cells showed significantly higher percent cells with nuclear localization of NP (Fig 3A, right panel and Fig 3D). To determine the amount of NP in the cells, we performed the kinetic analysis of NP expression at 6, 12, and 24 h p.i. by western blot assay and found that NP expression in A/X31-infected cell cultures treated with TG6-44 (50 μM) at 6 and 12 h p.i. was relatively decreased when compared to A/X31-infected cell cultures (Fig 4A). However, as shown in Fig 4B, virus titer analysis in supernatants collected at 24 h p.i. from cell cultures treated with TG6-44 failed to show any significant difference in virus titer when compared with vehicle control. Moreover, the decrease in NP expression and lack of changes in virus titer were also evident when A/X31 and TG6-44 were subjected to pre-incubation for 10 minutes prior to infecting THP-1 cells (Fig 4C). Likewise, reduction in NP expression was seen when TG6-44 treatment was delayed by 2 h after infection (Fig 4C). However, the consequent virus titer by 24 h p.i did not change significantly with either pre-incubation of TG6-44 with A/X31 or with the delayed treatment regimen (Fig 4D). Besides, analysis of IFN-β, an early anti-viral cytokine, in the virus infected cells failed to show any significant changes in IFN-β mRNA (Fig 5A) as well as protein (Fig 5B) at 6 and 12 h time points. However, at 24 h p.i., both IFN-β mRNA and protein levels in the TG6-44 treated cells were significantly reduced when compared to vehicle control (Fig 5A and 5B).

### TG6-44 treatment decreases virus-induced cell death in influenza A virus-infected cells

**Fig 6:**
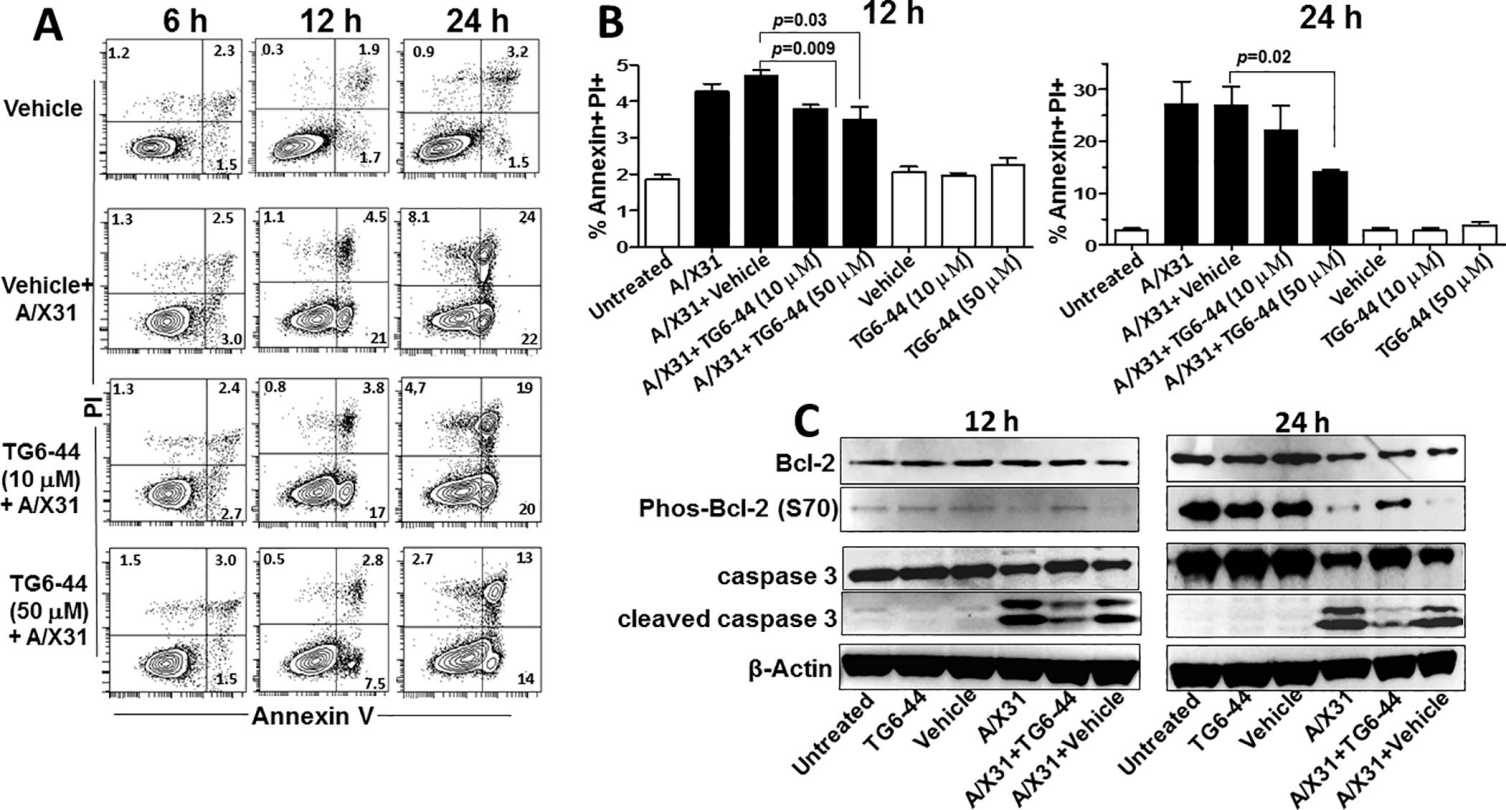
TG6-44 treatment leads to decrease in A/X31-induced cell death in THP-1 cells. A & B: A/X31-infected THP-1 cells treated with vehicle and/or TG6-44 were analyzed for percent annexin V^+^ and PI^+^ cells at 6, 12, and 24 h p.i. Representative FACS plots (A) and accumulative data from three independent experiments (B) are shown. Values in panel B represent percent mean ± SEM. C: Cell lysates from A/X31-infected THP-1 cells treated with vehicle and/or TG6-44 (50 μM) were assayed for Bcl-2, phosho Bcl-2, caspase-3, cleaved caspase-3, and β-actin, by western blot assay, as described in Materials and Methods. Data represent results from one of three independent experiments.

Marked suppression of A/X31-induced inflammatory mediators at early points (6 h and 12 h p.i) by treatment with TG6-44 raises the possibility that TG6-44 induces cell toxicity and increases cell death, especially in cultures treated with higher concentration of TG6-44 (50 μM). To address this, we determined percent cell death in A/X31-infected and/or TG6-44-treated THP-1 cell cultures by staining cells for an early (Annexin V) and a late (PI) marker of apoptosis. As shown in Fig 6A and 6B, when compared with vehicle treatment, cells infected with A/X31 demonstrated a significantly increased frequency of cell death by 12 h p.i.; by 24 h p.i., a higher percentage of cells. However, addition of TG6-44 at either 10 or 50 μM led to a significant decrease in cell death both at 12 h and 24 h p.i. (Fig 6A and 6B). As shown in Figs 6B and S1, the percentages of cell death for both concentrations of TG6-44 in 12 h and 24 h cell cultures in the absence of A/X31 infection was comparable to uninfected controls with or without vehicle. When PBMC were used for A/X31 infection, TG6-44 treatment (50 μM) led a moderate decrease in the percent cells that died (percentage of Annexin V^+^ PI^+^ cells) when compared to A/X31-infected cultures by 24 h p.i. (S3A Fig). Interestingly, detailed analysis of the PBMC subsets within the TG6-44 treated PBMC population revealed that the monocyte population (HLA-DR^+^, CD14^+^), a key cell type previously shown to be susceptible for influenza A virus infection [33, 34], showed the greatest rescue from virus-induced cell death (a decrease in percentage of Annexin V^+^ PI^+^ cells) (S3B Fig).

### TG6-44-induced decrease in cell death in A/X31-infected THP-1 cells is accompanied by modifications in pro-and anti-apoptotic molecules

TG6-44 treatment of A/X31-infected cells (THP-1 and PBMC) resulted in marked suppression of virus-induced inflammatory mediators and a significant decrease in virus-induced cell death (Figs 2, 6, S2 and S3). Previous studies of influenza A virus infection and virus-induced cell death have demonstrated increase of pro-apoptotic as well as a reduction of anti-apoptotic molecules in several cell types [35–38]. To determine whether the decrease in virus-induced cell death in cultures treated with TG6-44 was associated with modifications in anti-apoptotic and pro-apoptotic molecules, we performed a gene profiling assay (PCR array) using naïve THP-1 cells, THP-1 cells infected with A/X31, and A/X31-infected THP-1 cells treated with 50 μM TG6-44 at 12h p.i. We compared the fold induction of key anti-apoptotic (BCL2, BCL2L10, BIRC2, MCL-1, and XIAP) [39–41] and pro-apoptotic (CASP10, CASP3, and CASP5) [42] molecules in A/X31-infected as well as TG6-44-treated conditions with levels detected in naïve THP-1 cells. Consistent with the Annexin V/PI data (Fig 6A and 6B), A/X31-infected cell cultures treated with TG6-44 (50 μM) contained higher mRNA levels of anti-apoptotic and lower mRNA levels of pro-apoptotic molecules (S1 Table). Western blot analysis for phopho-Bcl-2 and cleaved caspase-3, two well established indicators of cell survival and cell death [36, 38], were used to confirm this finding. A/X31-infected cell cultures treated with TG6-44 had higher levels of phospho-Bcl-2 compared to A/X31-infected cell cultures (Fig 6C, upper panel), particularly at 24 h p. i. Also, when compared to A/X31-infected cells, TG6-44-treated cells infected with A/X31 demonstrated lower levels of cleaved caspase-3 both at 12 h and 24 h p.i. (Fig 6C, middle panel).

## Discussion

Expression of ROS and ROS-generating enzymes in different cell types is associated with boosting inflammation in tissues [5, 6]. Studies addressing the role of ROS in influenza A virus infection have established a link between ROS-generating enzymes and severity of disease caused by the virus [9, 12, 43]. In these studies, investigators used either mice deficient in ROS-generating enzymes or treatment with ROS-inhibitors for suppressing virus-induced ROS. In our study, we demonstrated that ROS levels are enhanced by influenza A virus infection, and that TG6-44, a novel quinazolin-derived small molecule, suppresses virus-induced ROS, inflammatory mediators, and virus infectivity in cell culture models of influenza A virus infection. Moreover, TG6-44 markedly reduced virus-induced cell death in treated cell cultures. Notably, TG6-44-mediated inhibition of virus-induced ROS, suppression of virus-induced inflammatory mediators and virus induced cell death was seen with both THP-1 cells as well as PBMC.

Previously, many studies have addressed the impact of ROS generation in different cell types and, more importantly, the consequence of its inhibition on health and disease (reviewed in [44, 45]). In the context of influenza A virus infection and ROS, Oda et al. showed that influenza A virus (A/Kumamoto/Y5/67/H2N2)-infected mice treated with pyran polymer conjugated SOD (O_2_^-^ scavenger) demonstrated significantly higher survival and reduced pathology in the lung tissue [11]. Vlahos et al. used apocyanin, a pharmacological agent that scavenges ROS, to prevent Nox2 activity in influenza A virus-infected mice. In this study, apocynin treatment, by virtue of its ability to prevent the association of p47phox with Nox2 subunit, led to a reduction in airway cellularity, superoxide level, and lung viral titer [12]. Moreover, Yamatz et al. used ebselen, a glutathione peroxidase (family of enzymes that catalyze the reduction of H_2_O_2_ into water and oxygen) mimetic, and found that ebselen treatment of influenza A virus (A/X-31/H3N2)-infected mice leads to reduction in lung mRNA levels of inflammatory mediators and proteases without any significant effects on either virus titer or virus-induced morbidity [8]. With reference to highly pathogenic influenza viruses and ROS, Perrone et al. used a NOS inhibitor (NG-monomethyl-l-arginine) and observed a delay in A/H1N1/1918 virus-induced morbidity and mortality [14]. Also, in an *in vitro* model of A/H5N1 influenza using infection, glycyrrhizin treated A549 cells showed a decrease in virus-induced ROS, inflammatory gene expression, as well as virus replication [15]. In the current experiments, we evaluated a quinazolin derivative (TG6-44) [23] for ROS inhibition in an *in vitro* model of influenza A virus infection. Based on our screening of TG6-44 for cytotoxicity on THP-1 cells (S1 Fig) and earlier investigations, by other groups, on different ROS-inhibitors [46, 47], we used two different concentrations (10 and 50 μM) of TG6-44 for evaluating its effects on virus infectivity as well as virus-induced inflammatory mediators and virus-induced cell death. We used SOD and DPI treatment of cell cultures as positive controls for ROS inhibition and, as expected, ROS levels decreased significantly in infected cells treated with DPI, an inhibitor of flavoenzymes, or by SOD, an enzyme that catalyzes O_2_^-^ into oxygen and hydrogen peroxide [29, 30]. Since SOD is a non-specific flavin-containing protein inhibitor, further studies using genetic approaches and specific NOX inhibitors are needed to define the specific sources of ROS modulated by MPO.

Since monocytes differentiate to phagocytes [48, 49] and are permissive for productive infection by influenza A virus, we chose to address expression levels and consequences of ROS inhibition both in a monocytic cell line (THP-1) and PBMC. Reports from earlier work, including our own, suggest that influenza infection of PBMC largely impacts the monocyte subset leading to productive infection and secretion of inflammatory mediators [33, 34]. Our current experiments demonstrate the anti-inflammatory effects of TG6-44 on host response to influenza A virus infection. Interestingly, addition of TG6-44 to THP-1 cell culture also resulted in decreased NP expression at early time points (Figs 3 and 4). To test whether the decrease in NP expression by TG6-44 treatment was the result of direct interference with virus infectivity by TG6-44, we pre-incubated TG6-44 with A/X31 prior to infection and then carried out infection of THP-1 cells. Also, to minimize the possibility of direct interference of TG6-44 on virus infection, we infected THP-1 cells with A/X31 and delayed TG6-44 treatment by 2 h. In both cases (Fig 4C), we found decreased levels of NP expression up to 12 h post infection. However, irrespective of the time point or sequence when TG6-44 was added to THP-1 cells, by 24 h p.i., there was no significant impact of TG6-44 treatment on virus titer. Also, analysis of cell cultures for IFN-β, a key anti-viral cytokine, showed no differences at 6 and 12 h p.i. However, by 24 h p.i., the levels of IFN-β were reduced in A/X31-infected cell cultures treated with TG6-44. Since NP expression was low irrespective of whether TG6-44 treatment was done along with or after virus infection but yet there was no major effect on key antiviral cytokine (IFN-β), it is possible that TG6-44 in some way interferes with virus entry and may not cause any alteration in anti-viral state in the cells. Moreover, the negative impact on IFN-β level by 24 h p.i. could be due to the suppressive effect of ROS inhibition on virus-induced cytokines (Fig 2). Notably, *in vivo* studies using Nox2-deficient mice [12, 43] as well as studies using pharmacological agents known to suppress ROS [12, 31] demonstrated reduction in lung virus titer. However, in the current study, TG6-44 mediated targeting of virus-induced ROS and inflammatory mediators does not seem to negatively impact the virus infectivity in *in vitro* model of influenza A virus infection.

Both influenza A virus infection as well as ROS production predisposes cells toward cell death [33, 50]. We found that TG6-44 treatment led to a decrease in A/X31-induced cell death in THP-1 cells and PBMC. Interestingly, monocytes, a key cell type previously shown to be susceptible for influenza virus infection [34], were less susceptible to cell death in presence of TG6-44. In fact, in response to TG6-44 treatment, the decrease in virus-induced cell death in 12 and 24 h cultures may have contributed toward the observed increase in IFN-γ, MCP-1, and IP-10. Reduction in cell death may be attributed to decreased level of virus-induced ROS in the presence of TG6-44 since higher levels of ROS are known to enhance cell apoptosis [50]. It is also possible that TG6-44 interferes with virus infectivity as suggested by our experiments where NP expression was decreased irrespective of time and sequence when TG6-44 was added. Consequently, the reduction in virus infectivity could have contributed towards decrease in cell death. Yet another, although unlikely, possibility is that TG6-44 binds to virus thereby decreasing infectivity at early time points.

The link between up-regulation of ROS and influenza A virus induced pathology [9, 43] raises the possibility and potential for evaluation of specific inhibitors that can suppress lung inflammation. However, experimental data in support of therapeutic role for ROS scavengers and antioxidants in the context of influenza virus infection and lung inflammation are necessary to support such a possibility. Further studies directly comparing the effects of TG6-44 treatment with ROS scavengers and antioxidants could support a therapeutic role for scavengers on suppression of influenza virus-induced overt cellular responses. Studies addressing the anti-tumor potency of quinazolin derivatives have established anti-proliferative and anti-angiogenic properties by their ability to target epidermal growth factor receptor and platelet derived growth factor receptor [17]. Our earlier studies with TG6-44 showed ROS inhibition in MPO-specific manner [23] highlighting the possibility that modulation of MPO in cells could alter the course of host response to influenza infection. In fact, Sugamata et al. established a role for phagocyte-derived MPO in early phase of influenza infection in mice [51]. Notably, MPO is involved in multiple pathways in different cell types and its role is not limited only to regulation of ROS production. For example, MPO treatment is shown to activate ERK/Akt pathway, p38 MAPK activation and enhanced nuclear translocation of NF-κB [52, 53]. In our studies, although treatment with TG6-44 was effective in suppressing virus infectivity and virus-induced inflammation in our *in vitro* cell culture model, the specific signaling pathways leading to ROS inhibition and other observed effects are not clear. While both validation of TG6-44 treatment on MPO-mediated ROS inhibition as well its effect on the course of influenza infection in an in vivo model remain as the immediate goal of our investigations, it is however evident from our current findings that the strategy of MPO inhibition using quinazolin derivatives such as TG6-44 may be helpful in diminishing immune mediated injury seen during influenza A virus infections. This may be particularly applicable to situations, including that with highly pathogenic influenza virus infections such as avian H5N1, where antivirals are effective in limiting the virus loads but immune injury caused by overt host response necessitates adjunct therapeutic modalities. It remains to be seen whether the anti-infective and anti-inflammatory properties of TG6-44 allow it to be used alone or in combination with currently available antivirals to effectively suppress the disease severity caused by influenza A virus infection of varying subtypes in *in vitro* and in *in vivo* models.

## Supporting information

S1 Fig TG6-44 treatment does not enhance cytotoxicity in uninfected THP-1 cells. Untreated, TG6-44-treated, or A/X31-infected THP-1 cells were analyzed for percent Annexin V^+^ and PI^+^ cells at 6, 12, and 24 h p.i. Representative FACS plots from 6, 12, and 24 h p.i. are shown. Values represent percent cells positive for Annexin V and/or PI. Data represent results from one of three independent experiments.
(TIF)

S2 Fig TG6-44 treatment leads to suppression of A/X31-induced inflammatory mediators. Cell culture supernatants from A/X31-infected THP-1 cell (A) and PBMC (B) treated with vehicle and/or TG6-44 were harvested and assayed for inflammatory mediators, by Bio-Plex assay, as described in Materials and Methods. Data represent results from one of three independent experiments. Values represent mean ± SEM.
(TIF)

S3 Fig TG6-44 treatment leads to decrease in A/X31-induced cell death in PBMC. A/X31-infected PBMC treated with vehicle and/or TG6-44 were analyzed for percent Annexin V^+^ and PI^+^ cells at 12 h and 24 h p.i. Representative FACS plots for PBMC from a single donor at 12 and 24 h p.i (A) and for monocyte population from two different donors at 24 h p.i. (B) are shown. Data under 3A represents results from one of three independent experiments (three donors). Values represent percent cells positive for Annexin V and/or PI.
(TIF)

S1 Table Gene expression of key cell survival and cell death associated molecules in THP-1 cells infected with A/X31. Cell lysates from A/X31-infected THP-1 cells treated with TG6-44 were harvested at 12 h p.i. and analyzed for cell survival and cell death associated genes by PCR array, as described under Materials and Methods. Values represent mRNA-fold change over uninfected controls. Data represent results from one of three independent experiments.
(DOCX)

S1 Raw images (PDF)
