# Supplementary figures and images for: Quinazolin-derived myeloperoxidase inhibitor suppresses influenza A virus-induced reactive oxygen species, pro-inflammatory mediators and improves cell survival
Source: PLoS One. 2021 Jul 19;16(7):e0254632. doi: 10.1371/journal.pone.0254632 (PMC8289044; doi:10.1371/journal.pone.0254632)

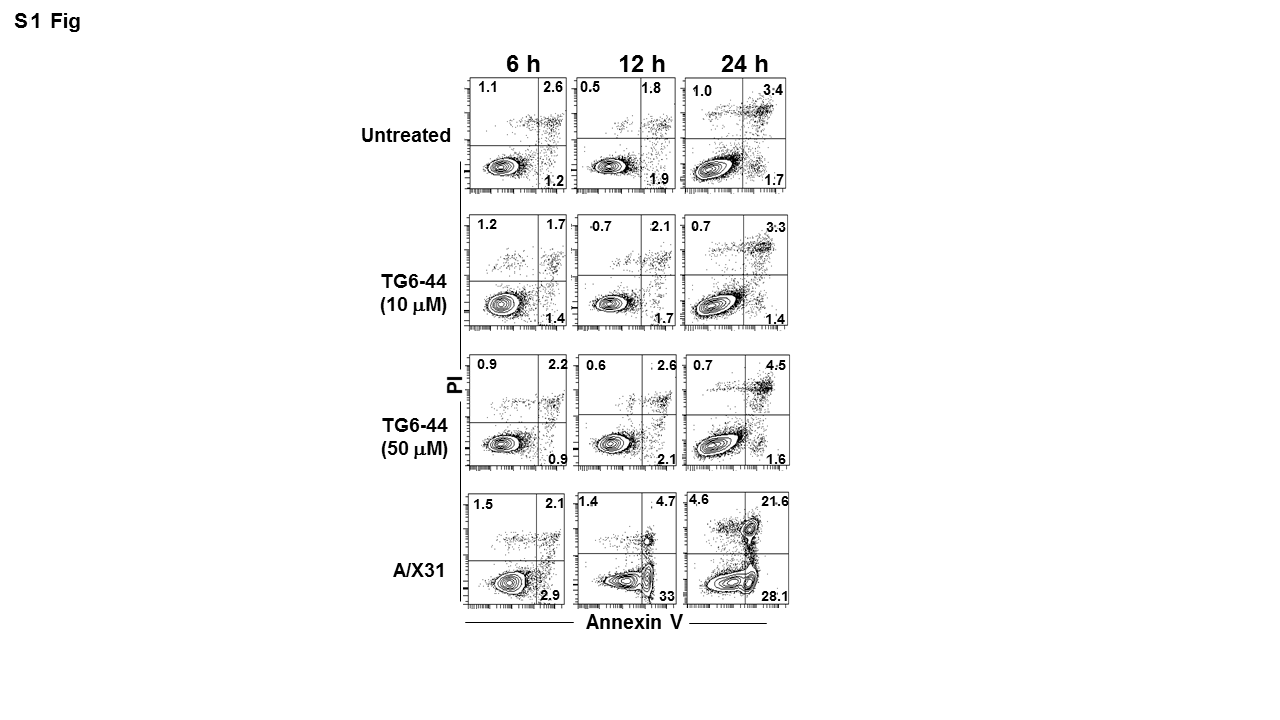

Supplement: S1 Fig — Untreated, TG6-44-treated, or A/X31-infected THP-1 cells were analyzed for percent Annexin V+ and PI+ cells at 6, 12, and 24 h p.i. Representative FACS plots from 6, 12, and 24 h p.i. are shown. Values represent percent cells positive for Annexin V and/or PI. Data represent results from one of three independent experiments. (TIF) [file pone.0254632.s001.tif]

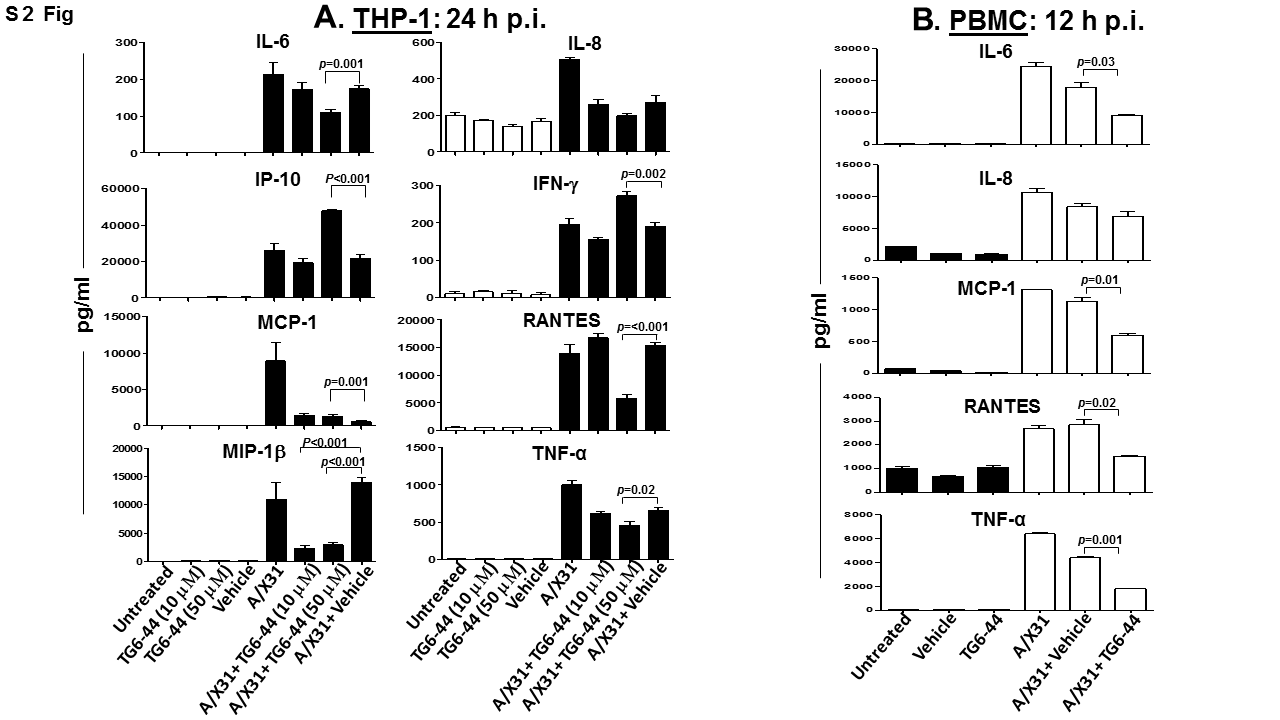

Supplement: S2 Fig — Cell culture supernatants from A/X31-infected THP-1 cell (A) and PBMC (B) treated with vehicle and/or TG6-44 were harvested and assayed for inflammatory mediators, by Bio-Plex assay, as described in Materials and Methods. Data represent results from one of three independent experiments. Values represent mean ± SEM. (TIF) [file pone.0254632.s002.tif]

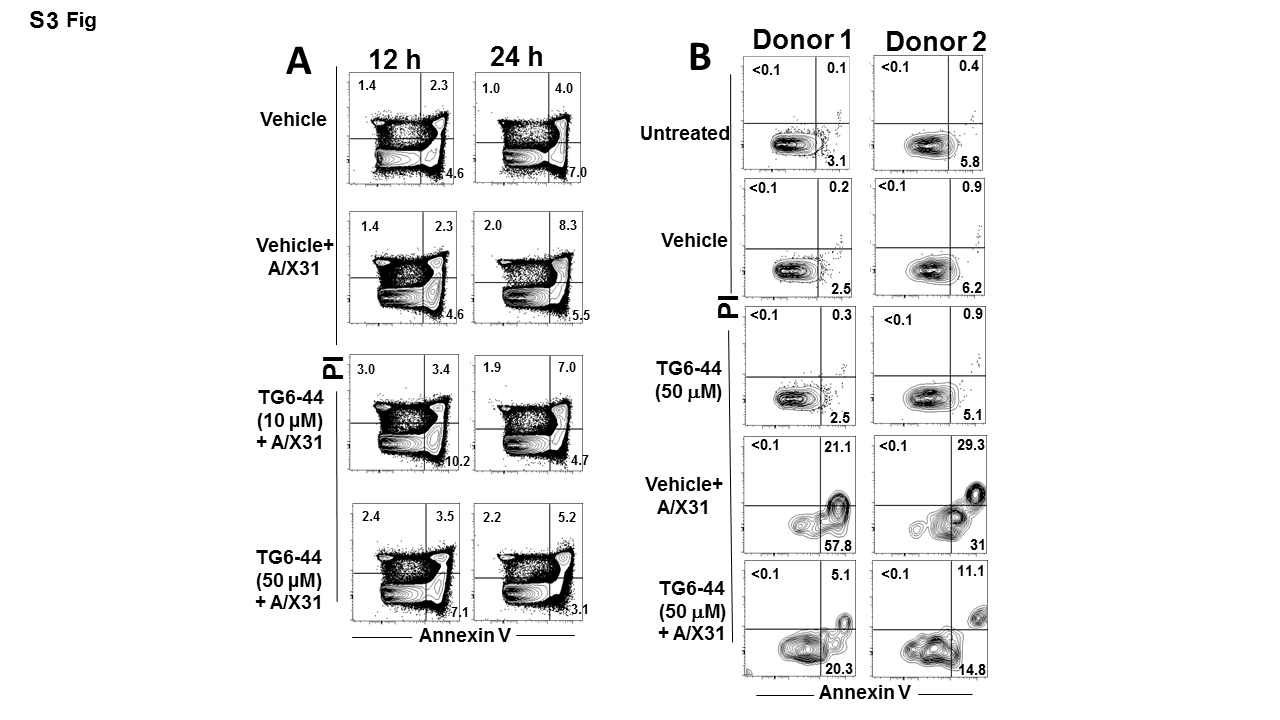

Supplement: S3 Fig — A/X31-infected PBMC treated with vehicle and/or TG6-44 were analyzed for percent Annexin V+ and PI+ cells at 12 h and 24 h p.i. Representative FACS plots for PBMC from a single donor at 12 and 24 h p.i (A) and for monocyte population from two different donors at 24 h p.i. (B) are shown. Data under 3A represents results from one of three independent experiments (three donors). Values represent percent cells positive for Annexin V and/or PI. (TIF) [file pone.0254632.s003.tif]
