# Supplementary material for: Quinazolin-derived myeloperoxidase inhibitor suppresses influenza A virus-induced reactive oxygen species, pro-inflammatory mediators and improves cell survival
Source: PLoS One. 2021 Jul 19;16(7):e0254632. doi: 10.1371/journal.pone.0254632 (PMC8289044; doi:10.1371/journal.pone.0254632)
Supplement: S1 Table — Cell lysates from A/X31-infected THP-1 cells treated with TG6-44 were harvested at 12 h p.i. and analyzed for cell survival and cell death associated genes by PCR array, as described under Materials and Methods. Values represent mRNA-fold change over uninfected controls. Data represent results from one of three independent experiments. (DOCX) [file pone.0254632.s004.docx]

**S1 Table**: Gene expression of key cell survival and cell death associated molecules in THP-1 cells infected with A/X31. Cell lysates from A/X31-infected THP-1 cells treated with TG6-44 were harvested at 12 h p.i. and analyzed for cell survival and cell death associated genes by PCR array, as described under Materials and Methods. Values represent mRNA-fold change over uninfected controls. Data represent results from one of three independent experiments.

| **Gene Symbol** | **A/X31** | **A/X31 +**  **TG6-44** | **Gene function** |
| --- | --- | --- | --- |
| BCL2 | 0.18 | 28.3 | Anti-apoptotic |
| BCL2L10 | 9.2 | 43.8 | Anti-apoptotic |
| BIRC2 | 5.5 | 69.3 | Anti-apoptotic |
| MCL-1 | 8.0 | 19.9 | Anti-apoptotic |
| XIAP | 2.8 | 43.8 | Anti-apoptotic |
| CASP10 | 15.0 | 7.3 | Apoptosis |
| CASP3 | 3.3 | 1.1 | Apoptosis |
| CASP5 | 47.9 | 30.4 | Apoptosis |
