# Supplementary material for: Quinazolin-derived myeloperoxidase inhibitor suppresses influenza A virus-induced reactive oxygen species, pro-inflammatory mediators and improves cell survival
Source: PLoS One. 2021 Jul 19;16(7):e0254632. doi: 10.1371/journal.pone.0254632 (PMC8289044; doi:10.1371/journal.pone.0254632)
Supplement: S1 Raw images — (PDF) [file pone.0254632.s005.pdf]

S1 Raw Images

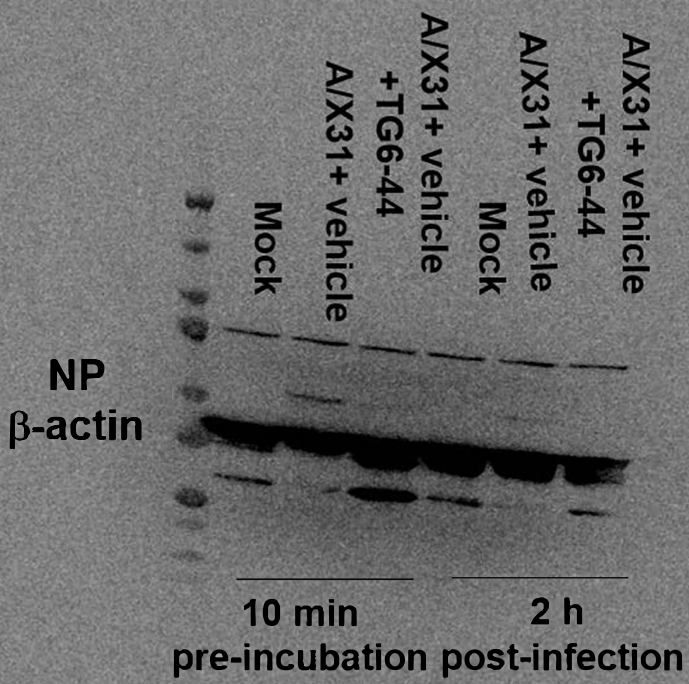

Figure 4, C, 6 hr

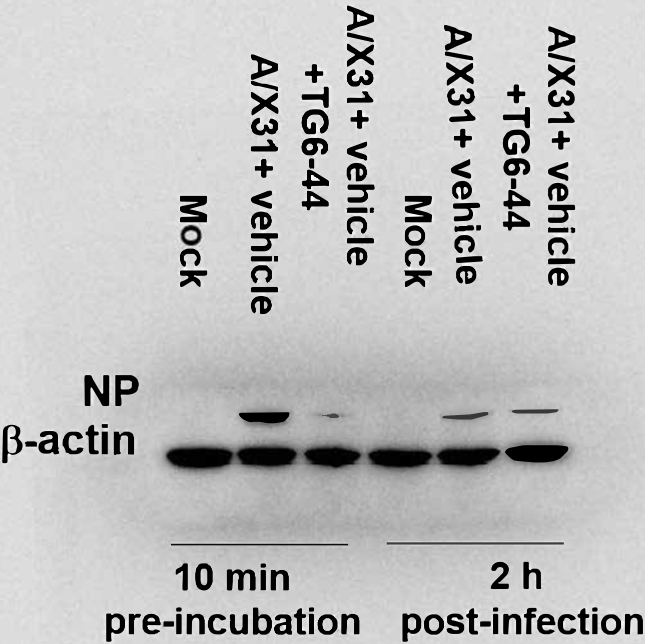

Figure 4, C, 12 hr

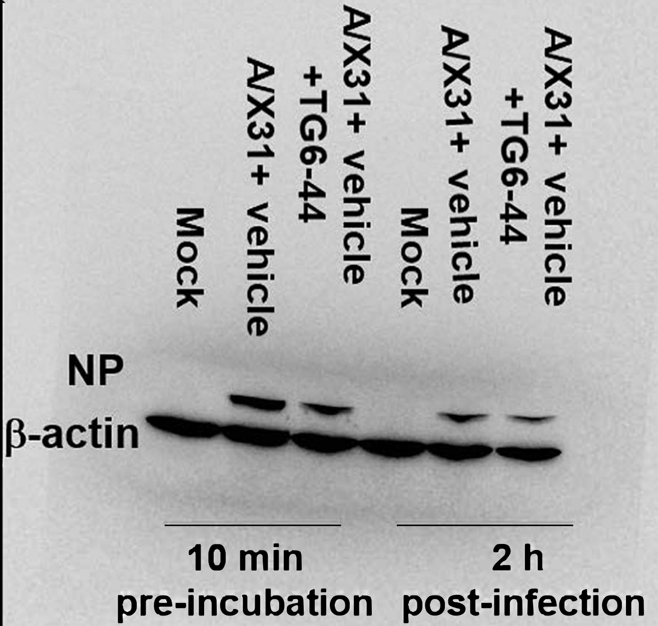

Figure 4, C, 24 hr

Phos-Bcl-2 (S70)

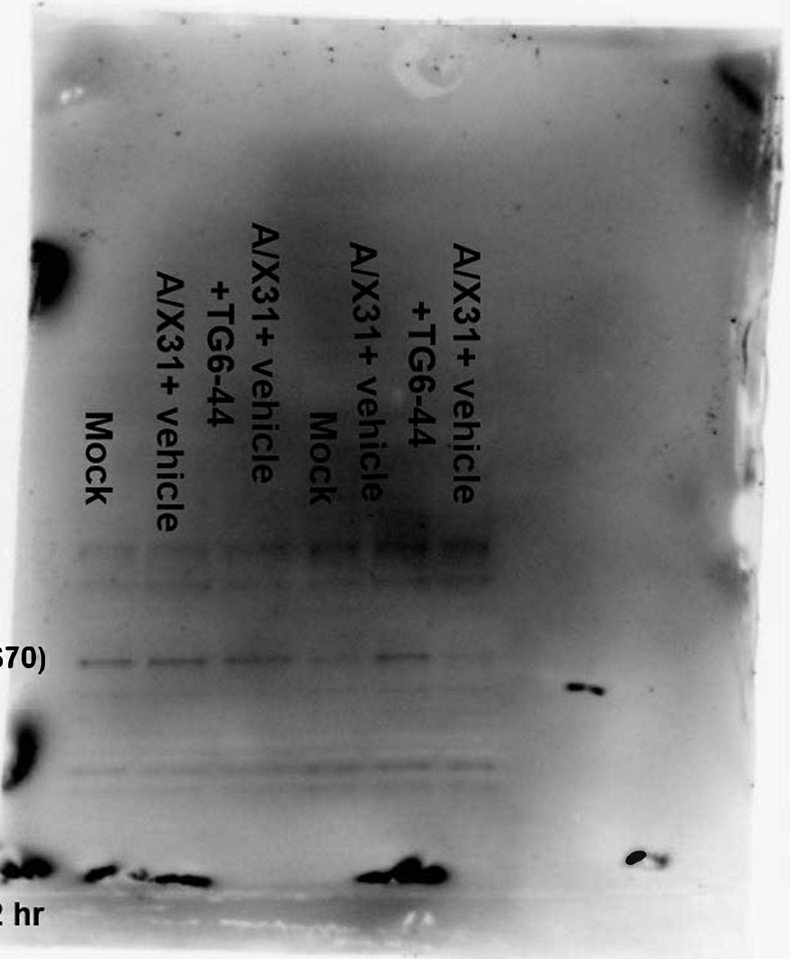

Figure 6, C, 12 hr

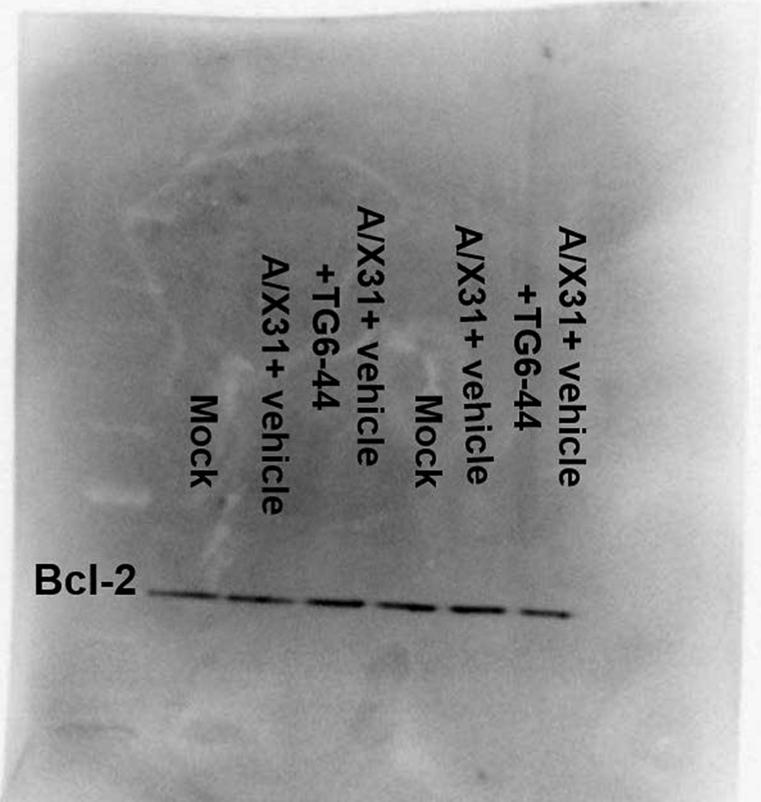

Figure 6, C, 12 hr

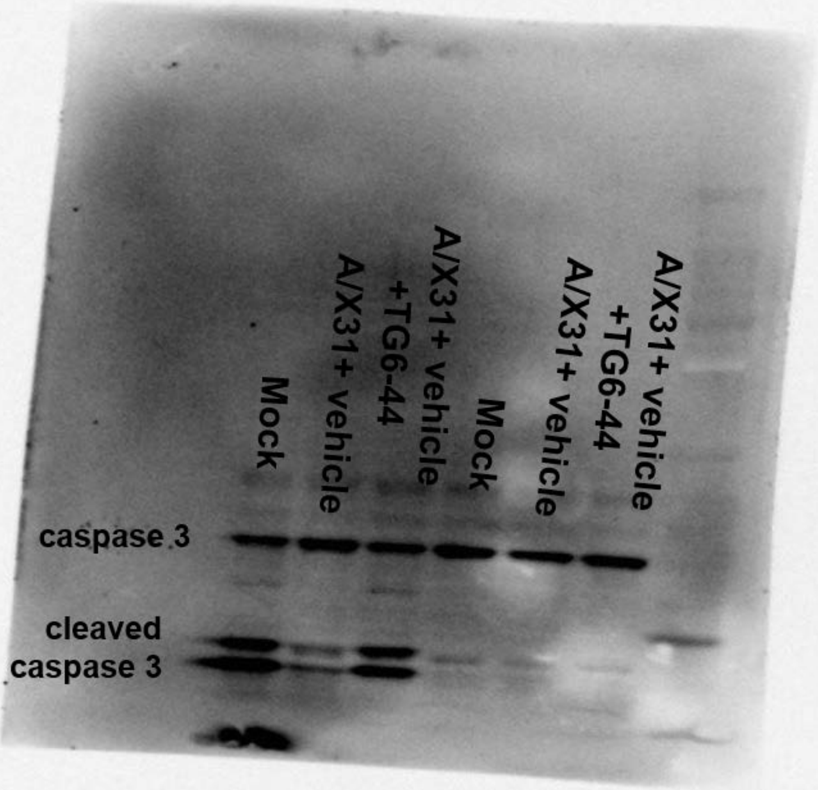

Figure 6, C, 12 hr
